# Elucidation of the Hydration Reaction of UHPC Using the PONKCS Method

**DOI:** 10.3390/ma13204661

**Published:** 2020-10-19

**Authors:** Hyunuk Kang, Nankyoung Lee, Juhyuk Moon

**Affiliations:** 1 Department of Civil and Environmental Engineering, Seoul National University, 1 Gwanak-ro, Gwanak-gu, Seoul 08826, Korea; kanghu93@snu.ac.kr (H.K.); nankyoung@snu.ac.kr (N.L.); 2 Institute of Construction and Environmental Engineering, Seoul National University, 1 Gwanak-ro, Gwanak-gu, Seoul 08826, Korea

**Keywords:** hydration reaction, nuclear magnetic resonance, thermogravimetry, ultra-high-performance concrete, X-ray diffraction, calcium silicate hydrate

## Abstract

This study explored the hydration reaction of ultra-high-performance concrete (UHPC) by using X-ray diffraction (XRD), nuclear magnetic resonance (NMR), and thermogravimetric analysis (TGA) as analysis methods. The partial- or no-known crystal structure (PONKCS) method was adopted to quantify the two main amorphous phases of silica fume and C-S-H; such quantification is critical for understanding the hydration reaction of UHPC. The measured compressive strength was explained well by the degree of hydration found by the PONKCS method, particularly the amount of amorphous C-S-H. During heat treatment, the pozzolanic reaction was more intensified by efficiently consuming silica fume. After heat treatment, weak but continuous hydration was observed, in which the cement hydration reaction was dominant. Furthermore, the study discussed some limitations of using the PONKCS method for studying the complicated hydration assemblage of UHPC based on the results of TGA and NMR. Generally, the PONKCS method underestimated the content of silica fume in the early age of heat treatment. Furthermore, the structural evolution of C-S-H, confirmed by NMR, should be considered for more accurate quantification of C-S-H formed in UHPC. Nevertheless, PONKCS-based XRD could be useful for understanding and optimizing the material properties of UHPC undergoing heat treatment.

## 1. Introduction

Cement is a mixture of the crystalline phases of substances such as alite, belite, and aluminate. The mixture undergoes different hydration reactions that result in the various material properties of concrete. To elucidate the chemical reactions and understand the evolution of the material properties, characterization methods, such as X-ray diffraction (XRD) and thermogravimetric analysis (TGA), are used to quantify the mineral phases during the hydration reaction [1,2,3,4,5]. In TGA, quantitative analysis is possible only for specific phases, such as calcium hydroxide and calcium carbonate [6,7,8]. In contrast, XRD can provide quantitative information for most of the crystalline phases; however, analysis of the amorphous phases, which is critical for investigating the time-dependent phase assemblage of cement-based materials, is still problematic [9]. For example, if amorphous or less-crystalline materials, such as silica fume, ground granulated blast-furnace slag, or fly ash, are mixed with cement for specific purposes, accurate separation of the amorphous reaction product from the raw material is difficult [10,11].

Generally, the Rietveld refinement method (using an internal or external standard method) can be applied to quantify an amorphous phase using XRD [12,13]. Additionally, if the partial or no known crystal structure (PONKCS) method, which can simulate the intensity of the virtually generated amorphous pattern, is used, quantitative analysis of the amorphous phases is possible [14,15,16]. The internal or external standard method can resolve one unknown (i.e., amorphous content)—that is, the total amount of existing amorphous phases. In contrast, the PONKCS method can be theoretically applied to investigate the phase quantification of two or more different amorphous phases. This is a plausible approach because each amorphous phase has a distinct hump range, which can be individually fitted before the reaction occurs.

Ultra-high-performance concrete (UHPC) is a structural material that is implemented in construction [17,18,19,20]. Its durability is especially outstanding due to its excellent compressive strength and dense microstructure [21,22]. Typical high-strength concrete-like UHPC contains amorphous silica fume as a raw material, and its main hydration product is C-S-H, the main hydration product of general cement hydration. Since both the raw material and resulting main hydration product are in non-crystalline phases, the XRD-based characterization of the hydration mechanism of UHPC (e.g., the degree of consumption of the silica fume or production of C-S-H) is difficult [23]. Additionally, the lower degree of hydration of UHPC also makes accurate quantitative phase identification more difficult [13,24,25].

This study aimed to elucidate the hydration reactions of UHPC using the PONKCS method due to UHPC’s amorphous phase in the raw mixture (i.e., silica fume) and in hydrated form (i.e., C-S-H). By fitting both phases using the PONKCS method, accurate hydration assemblage of the complex system of UHPC was obtained. For example, it included the degree of consumption of silica fume and the production of C-S-H. In addition to the quantitative XRD (QXRD) analysis, TGA and nuclear magnetic resonance (NMR) analysis were also performed to verify the time-dependent hydration characteristics of UHPC. Finally, the study investigated the strength development of UHPC via interpretation by mineralogical characterizations.

## 2. Materials and Experimental Details

### 2.1. Sample Preparation

**Figure 1:**
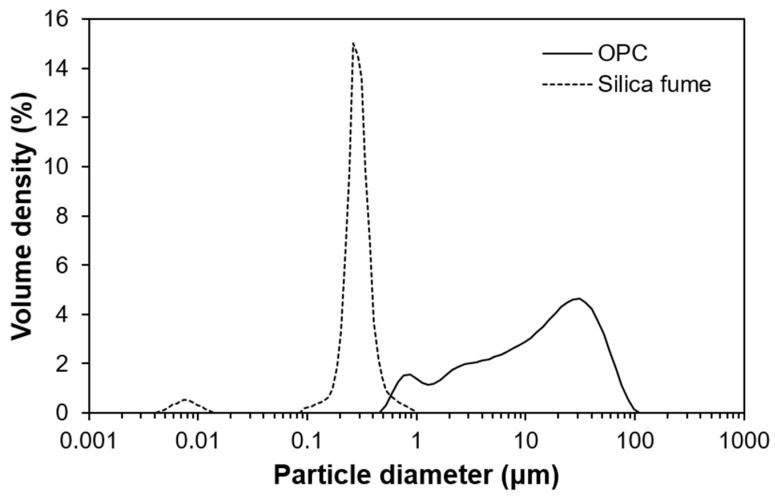
Particle size distributions of ordinary Portland cement and silica fume.

OPC (Ordinary Portland cement) (Union Cement Co., Ltd., Chungcheong-do, Korea), silica fume (Grade 940U, Elkem, Olso, Norway), silica powder (S-SIL 10, SAC, Ulsan, Korea), silica sand (Saeron Co., Ltd., Gangwon-do, Korea), and polycarboxylate ether-based superplasticizer (Flowmix 3000U, Dongnam, Gyeonggi-do, Korea) were used based on the authors’ previous UHPC studies [26,27,28,29]. The mix design used in this study is shown in Table 1. The particle size distributions of the OPC and silica fume (Figure 1) were obtained using a microparticle size analyzer (Malvern Instruments., LTD., Malvern, UK). To measure compressive strength, quartz powder and silica sand were included, as shown in Table 1. Meanwhile, quartz powder and silica sand were excluded in samples for XRD, TGA, and NMR because the components are chemically inert.

The paste and UHPC samples were prepared by dry-mixing for 1 min, followed by mixing with the polycarboxylate ether-based superplasticizer and water for 5 min. Then, they were placed into the 50 × 50 × 50 mm^3^ cubic mold and cured at room temperature for 1 day, and then cured at 90 °C for 2 days. Subsequently, they were cured at 20 °C until subsequent testing.

### 2.2. Experimental Details

To measure compressive strength, three identical cubes were tested to determine the average compressive strength of the specimens cured for 1, 2, 4, and 28 days. For XRD, TGA, and NMR measurements, the paste was soaked first in isopropyl alcohol and then ethyl ether to stop the hydration (by removing free water) at the target date of investigation (i.e., 1, 2, 4, and 28 days). The remaining ethyl ether was removed by heating the specimen at 40 °C.

**Figure 2:**
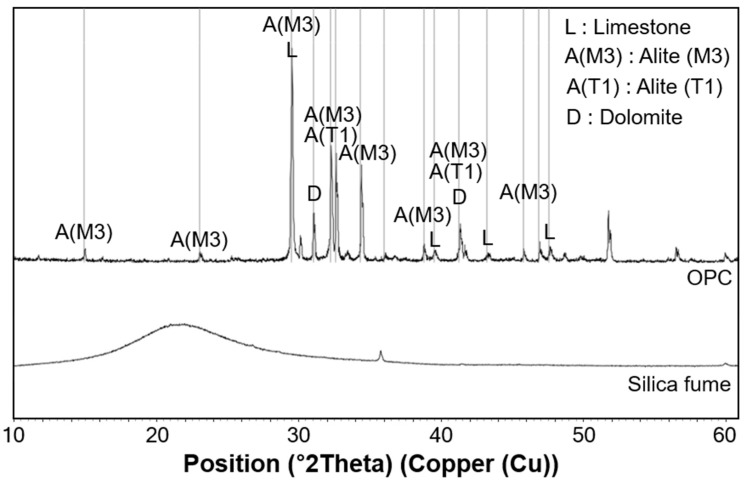
Measured X-ray diffraction patterns of raw materials.

An X-ray diffractometer (D2 Phaser, Bruker Co. Ltd., Land Baden-Württemberg, Germany) equipped with Cu-Kα radiation (λ = 1.5418 Å) was used to measure the powder’s XRD pattern in the range of 2θ between 5˚ and 60˚. The acquired diffraction patterns of the paste specimens were analyzed using the TOPAS software version 7.0 (Bruker Co. Ltd., Land Baden-Württemberg, Germany). The XRD results for the raw OPC and silica fume are presented in Figure 2. The main constituents of the OPC were alite (51.6%), belite (0.5%), dolomite (18.1%), and limestone (29.7%). A hump was observed for the silica fume between 14˚ and 30˚ [30].

TGA was performed in an N_2_ environment using a DSC/TG system (SDT Q600, TA Instrument Ltd., Newcastle, DE, USA) at a heating rate of 10 K/min up to 1050 °C. The mineral phases of calcium hydroxide and calcium carbonate were calculated based on their decomposition temperatures of 400–500 °C and 600–800 °C, respectively [12]. The ^29^Si MAS NMR experiments were performed using Advance III HD (Bruker, Karlsruhe, Germany) at 119.182 MHz. The NMR spectra were obtained using a 5-mm HX CPMAS probe and a 5-mm zirconia rotor with a rotation speed of 10.0 MHz, a pulse width of 2.2 μs, and a relaxation delay of 22 s. The ^29^Si chemical shifts referenced external samples of −135.5 ppm of tetramethyl silane (TMS) and −135.5 ppm of tetrakis silane to 0 ppm of aqueous AlCl_3_, respectively.

## 3. Experimental Results

### 3.1. Compressive Strength Results

**Figure 3:**
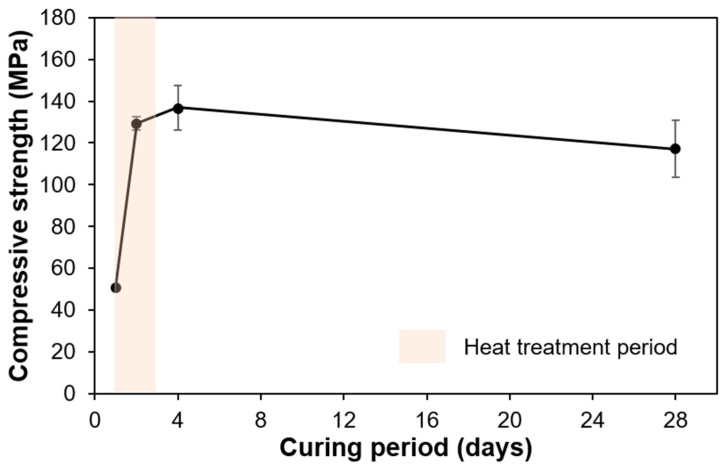
Compressive strength of the specimens.

The average and standard deviation of the compressive strengths are shown in Figure 3. The average compressive strengths cured for 1, 2, 4, and 28 days were 50.7, 129.3, 136.9, and 117.2 MPa, respectively. The strength significantly increased during the 2 days of the heat treatment period, and then it slightly decreased from 4 to 28 days.

### 3.2. TGA Results

**Figure 4:**
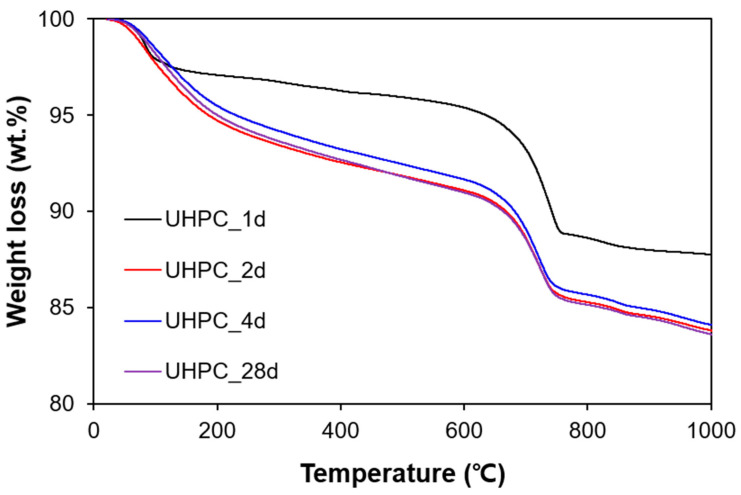
Measured TGA of all specimens.

The results of the TGA are shown in Figure 4. Regardless of curing time, calcium hydroxide (decomposition range between 400 and 500 °C) was hardly detected; this finding was similar to the authors’ previous hydration studies on UHPC [13,31,32]. The main weight loss occurred between 600 and 800 °C, which indicated the decomposition of calcium carbonate [33]. In this study, normalization to anhydrous was performed based on the following equation

(1) $$W_{\mathrm{corrected}}\;=\;\frac{W_{\mathrm{Rietveld}}}{1-H_{2}O_{\mathrm{Bound},\mathrm{TGA}}}$$

where W_corrected_, W_Rietveld_, and H_2_O_Bound,TGA_ indicate the normalized result, the weight percentage from XRD analysis, and the chemically bound water (CBW), respectively.

The CBW was calculated from the weight loss value at 600 °C. The CBW for 1, 2, 4, and 28 days of the samples was 4.6%, 8.9%, 8.3%, and 9.0%, respectively. Generally, the amount of CBW increased as the curing time increased; the CBW represented the degree of hydration. However, during the heat treatment period, the CBW was slightly reduced (from 8.9% to 8.3%), possibly because partial CBW in pre-formed, and formulating C-S-H can be lost at 90 °C [16,34].

### 3.3. XRD Results

**Figure 5:**
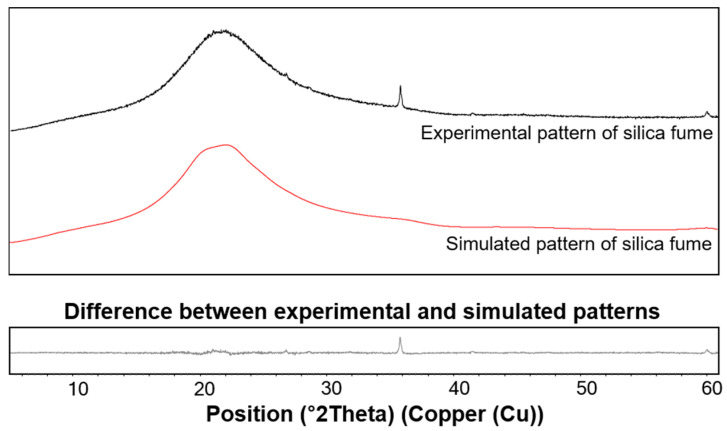
Experimental and simulated diffraction patterns of silica fume.

**Figure 6:**
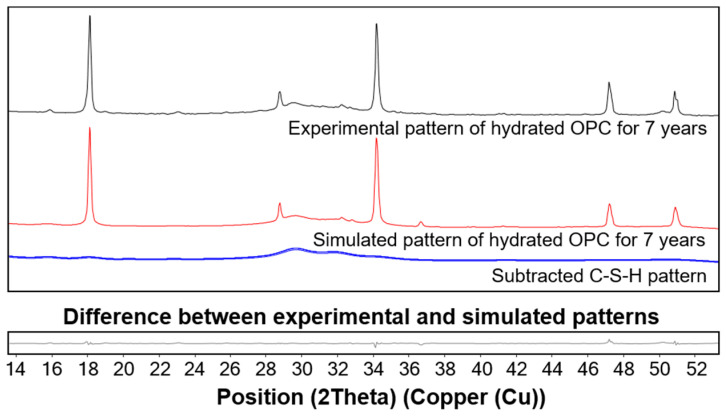
Experimental and simulated diffraction patterns of C-S-H. The experimental pattern of hydrated OPC for 7 years was reproduced from a previous study [23].

To use the PONKCS method for QXRD, a preliminary experiment was conducted on a mixture of silica fume and quartz powder. A sample mixed with a weight ratio of quartz powder to silica fume of 1:1 was prepared, and the database was generated as follows [35]

(2) $${\left(\mathrm{ZMV}\right)}_{\mathrm{Amorphous}}\;=\;\frac{W_{\mathrm{Amorphous}}}{W_{\mathrm{Quartz}}}\;\times \;\frac{S_{\mathrm{Quartz}}}{S_{\mathrm{Amorphous}}}\;\times \;{\left(\mathrm{ZMV}\right)}_{\mathrm{Quartz}}$$

where W_x_, S_x_, ZM, and V indicate the weight percentage of x, scale factor of x, cell mass, and unit cell volume, respectively. Based on this method, the scale factor, virtual Miller constant, and corresponding intensity of silica fume were probabilistically calculated to generate the hypothetical space group for the diffraction pattern of silica fume, and a similar method was used to generate the C-S-H pattern. The XRD pattern obtained from the OPC cured for 7 years was used after excluding peak contributions from the known crystalline phases [36]. Then, the fitted patterns of the silica fume and C-S-H were used for the quantitative phase analysis of the UHPC to evaluate the weight variation of the two amorphous phases. The virtual patterns of the silica fume and C-S-H generated with the PONKCS method are presented in Figure 5 and Figure 6, respectively.

For the PONKCS method and subsequent refinement processes, the background was excluded by using a polynomial function of an order less than 5. Previous studies indicated that inaccurate fitting may occur when a low angle range is included in the PONKCS method or Rietveld analysis [36]. In the present study, the refinement was performed from 8˚ 2θ degrees, considering the presence of ettringite. Then, to reduce scattering at a low angle, a slit was adopted to enhance the fitting accuracy. Neither the internal standard nor the external standard method was used in this study, as the pattern corresponding to the background and all humps would have been assigned to the weight of an amorphous phase, thereby significantly overestimating the amount of actual amorphous content in the samples. Furthermore, separating the phase content of the two main amorphous phases (i.e., silica fume and C-S-H) would not have been possible in UHPC hydration with either method.

**Figure 7:**
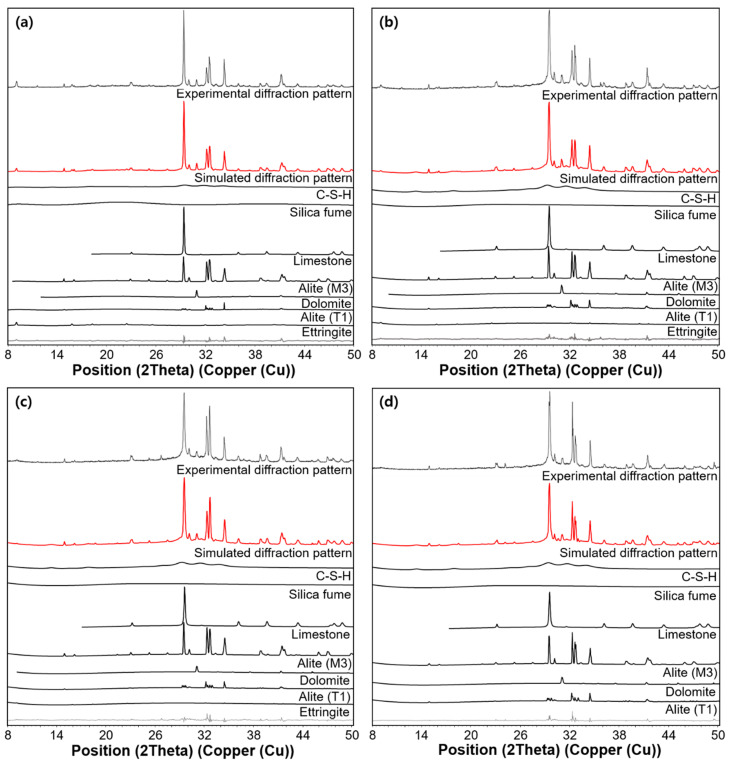
Rietveld refinement results of all samples: (**a**) UHPC_1 day, (**b**) UHPC_2 days, (**c**) UHPC_4 days, (**d**) UHPC_28 days.

**Figure 8:**
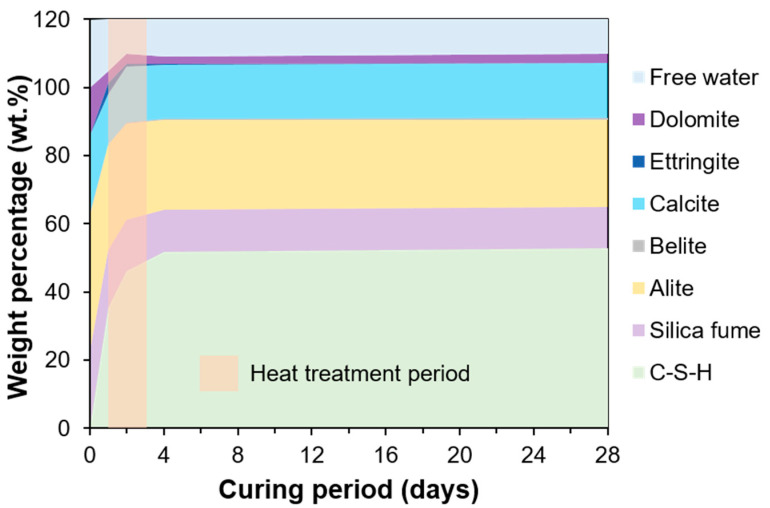
QXRD results after normalization to anhydrous content.

The measured XRD patterns (UHPC_1 day, UHPC_2 days, UHPC_4 days, and UHPC_28 days) and the corresponding refined patterns are shown in Figure 7. The results of the QXRD are shown in Figure 8. Note that the free water content in the Figure 8 was calculated by the subtraction of the scaled solid amount (using the CBW value at each date) from the initial mix proportion. The main hydration products were C-S-H and ettringite, while raw materials of silica fume, alite, limestone, and dolomite were still identified in all ages. However, minor phases in OPC, such as belite and aluminate, were not identified after hydration began. In the samples cured for 2 days and 4 days, ettringite was found at around 9˚, but it disappeared at 4 days, indicating that it was completely decomposed through curing at 90 °C for 2 days [37]. The reduction in the intensity of the peaks corresponding to alite indicated the continuous progress of hydration at all ages. After normalization to anhydrous content, the silica fume was measured as 16.9%, 15.1%, 12.3%, and 12.1%, while C-S-H was about 35.3%, 46.2%, 51.8%, and 52.8% in samples cured for 1, 2, 4, and 28 days, respectively. Thus, the quantitative separation of the two amorphous phases was possible based on the adopted PONKCS method.

### 3.4. NMR Results

**Figure 9:**
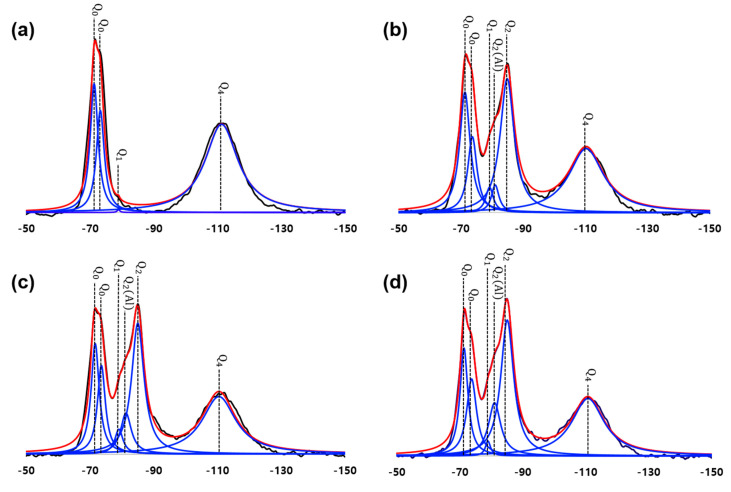
^29^Si magic angle spinning NMR (^29^Si MAS NMR) spectra and de-convoluted patterns: (**a**) UHPC_1 day, (**b**) UHPC_2 days, (**c**) UHPC_4 days, and (**d**) UHPC_28 days.

Figure 9 shows the deconvolution data from the NMR measurements. Similar to the results obtained with typical cement-based materials, a set of Q^0^ peaks (−71 and −74 ppm), a Q^1^ peak (−79 ppm), a Q^2^(1Al) peak (−81 ppm), a Q^2^ peak (−85 ppm), and a Q^4^ peak (−110 ppm) were identified. The peaks were deconvoluted using Gaussian and Lorentzian functions, and the results are presented in Table 2 [25,38,39]. The Q^0^ and Q^4^ peak represented alite and silica fume, respectively [39]. The intensity of both peaks generally decreased, while the intensity of the peaks corresponding to the C-S-H tended to increase as hydration progressed.

The Q^1^ peak represented the end of the silicate chain, while the Q^2^(1Al) and Q^2^ peaks represented the connected silicate tetrahedral chains of C-S-H. The length of the formulated silicate chain (i.e., the mean chain length (MCL)) can be estimated as follows [40]

(3) $$MCL_{nc}\;=\;\frac{2\left[Q^{1}+Q^{2}+\frac{3}{2}Q^{2}\left(1\mathrm{Al}\right)\right]}{Q^{1}}$$

(4) $$\frac{\mathrm{Al}}{{\mathrm{Si}}_{nc}}\;=\;\frac{\frac{1}{2}Q^{2}\left(1\mathrm{Al}\right)}{Q^{1}+Q^{2}+Q^{2}\left(1\mathrm{Al}\right)}$$

The estimated MCL increased as the hydration reaction progressed (Table 2). At 1 day, C-S-H with a small MCL was formed during the early stages of hydration. During the heat treatment, partial decomposition of C-S-H may have occurred (as determined by the reduced CBW in Figure 4); this could be associated with the water loss in the newly formed C-S-H at the perimeter of pre-existing C-S-H. Thus, the MCL and the fractal dimension of the C-S-H were significantly increased [13].

After the heat treatment period, continuous hydration of OPC could have still occurred, resulting in the higher stability of the formed C-S-H structure. Therefore, the crystallinity of C-S-H was relatively high at later ages. The estimated Al/Si ratio had a similar tendency to that of the MCL, as determined by Equation (4) [25]. However, more rigorous investigation must be performed to refine the equation for estimating the nanostructure of C-(A)-S-H, especially in restrained environments or for UHPC [13,40].

## 4. Discussion

### 4.1. C-S-H Formation Confirmed by XRD and TGA

This section discusses the C-S-H formation of UHPC upon curing based on the experimental results of XRD and TGA. The amount of C-S-H increased as hydration progressed, and the increase was the largest during the heat treatment process. TGA can be used to estimate the amount of C-S-H by measuring the weight loss of water in the C-S-H. However, the precise range of temperatures at which C-S-H decomposes (i.e., the evaporation of physically or chemically bound water) has not been fully clarified. For example, Taylor et al. reported the range as 115–125 °C, while Odelson et al. used the range of 200–400 °C to estimate the amount of water loss associated with C-S-H [41,42].

**Figure 10:**
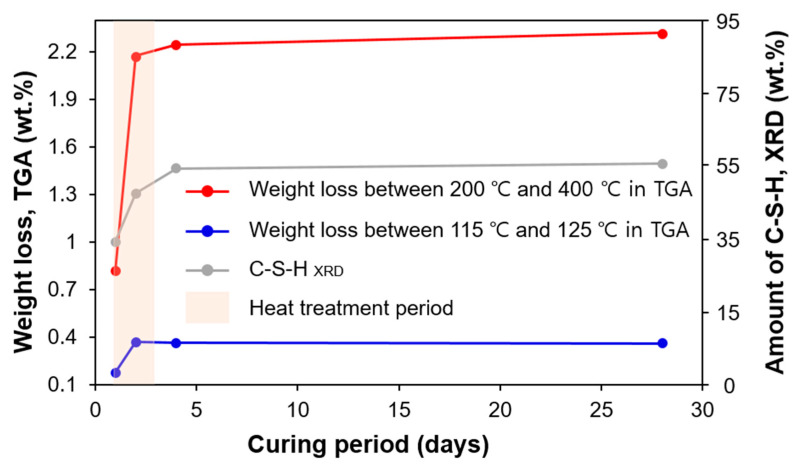
Comparison of C-S-H formation measured from XRD and TGA.

Figure 10 compares the variations of the measured amount of C-S-H from the PONKCS method and weight loss related to the C-S-H in the two different temperature ranges. The XRD yielded up to a C-S-H weight of 52% in the UHPC (excluding the weight contributions of quartz powder and silica sand) at 28 days. As expected, the net amount of formation of C-S-H was the largest during the heat treatment period. However, the potential error of the XRD-based quantification of the C-S-H was that the used C-S-H model was subtracted from the 7-year-old hydrated OPC. Thus, it was not simulated using the C-S-H pattern in UHPC, which might have been different considering the complex microstructure of UHPC due to heat treatment as well as the pozzolanic reaction [13].

According to the TGA results, the calculated weight loss in both temperature ranges quickly increased during the heat treatment period, and then stabilized. Interestingly, the absolute weight loss, as well as the degree of increase in 200–400 °C, was calculated to be much larger than that in the range from 115 to 125 °C. Weakly bound water in C-S-H may be easily lost during the heat treatment, so the weight loss measured after the heat treatment can be small. Even so, the trend measured by XRD analysis (i.e., the weight percentage of C-S-H) was similar to that found by TGA (i.e., weight loss of water in C-S-H).

### 4.2. Relationship between Compressive Strength and C-S-H Formation

**Figure 11:**
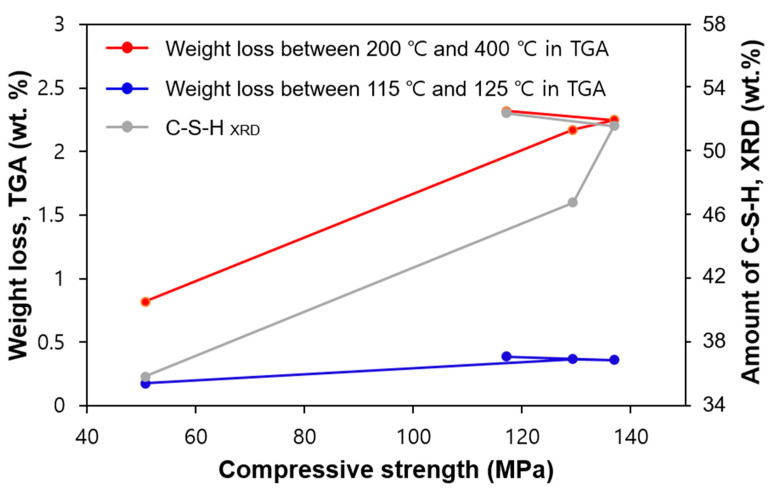
Relationship between strength development and the estimated amount of C-S-H.

The relationship between compressive strength and C-S-H formation in the samples cured for 1, 2, 4, and 28 days is shown in Figure 11. Except for the UHPC_28 days sample, the relationship between the development of strength and the amount of C-S-H was directly proportional. In the early stages of heat treatment, the produced amount of C-S-H was the greatest due to the acceleration of the hydration reaction caused by heat treatment. Thus, the compressive strength was also greatly enhanced [13,31]. However, the relationship between compressive strength and C-S-H in the samples of UHPC_4 days and UHPC_28 days was not clear. The main cause of this phenomenon was due to the slight strength reduction at 28 days along with a slightly increased production of C-S-H. As was previously explained, after heat treatment in UHPC, the available space for subsequent hydration is limited. Therefore, the later age of hydration in dense microstructures may cause micro-cracking in the matrix, thereby leading to a slight decrease in strength at later ages [31].

### 4.3. Relationship between Consumption of Silica Fume and Formation of C-S-H

**Figure 12:**
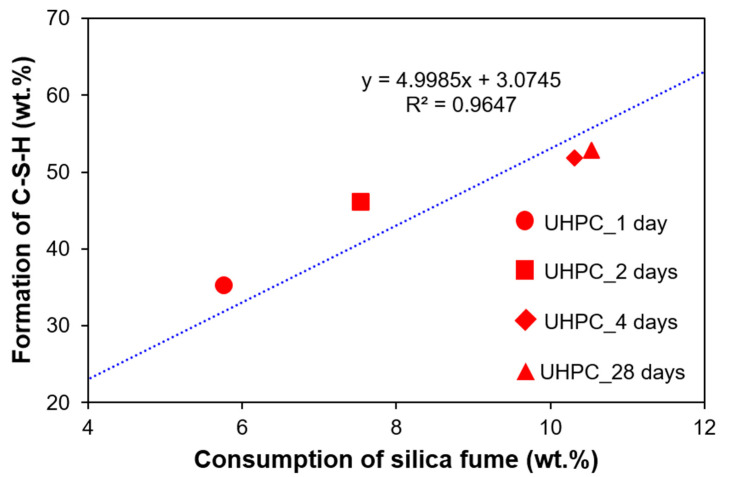
Relationship between consumption of silica fume and formation of C-S-H.

Figure 12 shows the relationship between the formation of C-S-H and the consumption of silica fume. First, the consumption of silica fume was the greatest during the heat treatment period, which was associated with the largest formation of C-S-H in that period (at 2 and 4 days). The consumption of silica fume can be explained by the pozzolanic reaction, which can be efficiently accelerated by the consumption of portlandite during heat treatment. This was also consistent with a recent hydration study of UHPC [13]. As a result of the enhanced pozzolanic reaction, the detected portlandite after the heat treatment was found to be less than 0.5 wt.%.

Heat treatment is the conventional curing method for UHPC to expedite the production in the precast form [24]. However, the degree of hydration in UHPC is very low, due to the limited amount of water and space availability [13,31]. In this study, silica fume was still identified at 28 days (Figure 9), while the portlandite was almost completely consumed during the heat treatment. Therefore, the pozzolanic reaction during heat treatment was mostly terminated due to the lack of portlandite. Further hydration after heat treatment was mostly based on the OPC hydration, which is not very efficient in terms of filling space via hydration reaction. While the pozzolanic reaction can occur in the surrounding hydration products, cement hydration at that stage requires the diffusion of water through pre-hydrated products. This subsequent hydration may induce micro-cracking in the matrix, thus slightly reducing the compressive strength.

As seen in Figure 12, the relationship between the formation of C-S-H and the consumption of silica fume was generally linear. Greater C-S-H formation at 1 and 2 days (points above the trend line) indicated the relatively higher OPC hydration (i.e., less consumption of silica fume but more formation of C-S-H). The points at 4 and 28 days lie below the trend line, showing greater consumption of silica fume compared to the produced amount of C-S-H. This analysis also confirmed that the pozzolanic reaction was intensified during the heat treatment period. However, it also may have suggested that the crystallinity between C-S-H due to OPC hydration and pozzolanic reaction can be different. This potential difference could impact the accuracy of fitting C-S-H with the PONKCS method, which only relied on the C-S-H pattern subtracted from OPC hydration at 7 years [36].

### 4.4. Comparison of the Reactivity of Silica Fume and Clinker Materials from XRD and NMR

**Figure 13:**
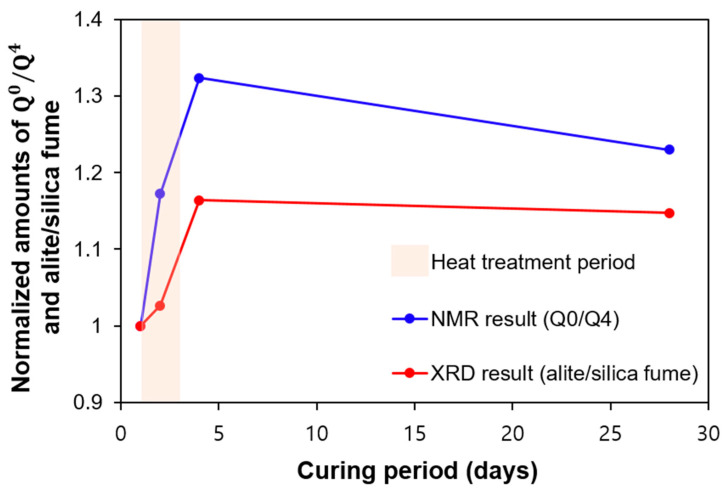
Normalized amounts of Q^0^/Q^4^ and alite/silica fume as a function of curing time.

As discussed, the potential difference in the crystallinity of the C-S-H formed by the pozzolanic reaction (mainly during heat treatment) and that formed by OPC hydration may affect the quantitative content of C-S-H. Furthermore, as confirmed by the NMR experiment, the nanostructure of C-S-(A)-H also evolved as the length of the MCL and Al/Si changed. The impact of the MCL and Al/Si on the crystallinity of C-S-H should be considered for more accurate quantification of the content of C-S-H [13]. Instead, the relative reactivity of silica fume and clinker (i.e., alite) can be seen in the NMR and QXRD results. Figure 13 shows the variation in the ratios of Q^0^/Q^4^, as found by NMR (Q^0^ and Q^4^ indicate alite and silica fume, respectively) and alite/silica fume, as found by QXRD. Except for the sample of UHPC_2 days, the similar tendency of an abrupt increase at early ages and a steady decrease at later ages was confirmed.

The abrupt increase in both Q^0^/Q^4^ and alite/silica fume directly indicated greater consumption of silica fume at early ages. Again, this can be related to the efficiently accelerated pozzolanic reaction due to heat treatment. Additionally, the steady decrease in those values at later ages can be interpreted as a more active OPC hydration (instead of the pozzolanic reaction). Although the general trend is similar, the NMR showed a higher value compared to XRD at 2 days due to the difference caused by the PONKCS method, which was adopted to quantify the silica fume in a mixture.

Compared to the measurement accuracy of NMR in identifying silica fume and alite, QXRD certainly had a limitation in accurately quantifying the relative amount of silica fume intrinsically, represented by a small hump in the XRD pattern. Although this study was able to capture the silica fume using the PONKCS method, the accuracy of quantifying the silica fume may have been lower, especially during the accelerated pozzolanic reaction period. During the heat treatment, the consumption of well-dispersed silica fume particles occurred first. Later, an additional reaction proceeded for the bulk type of silica fume, which had a stronger intensity in the XRD experiment. Therefore, during the early stages of heat treatment, the PONKCS method may underestimate the amount of silica fume.

## 5. Conclusions

This study elucidated the complicated hydration reaction of UHPC using various experiments. Using the PONKCS method, the amorphous phases of silica fume and C-S-H were successfully separated and quantified. To confirm the reliability of the QXRD results, TGA and NMR were also used. The conclusions of this study are as follows:

1. The compressive strength of UHPC cured for 1, 2, 4, and 28 days was 50.7, 129.3, 136.9, and 117.2 MPa, respectively. The 2 days of heat treatment significantly enhanced the pozzolanic reaction, effectively increasing the compressive strength. However, it showed a slight decrease at later ages, which was explained by the micro-cracking that may have been caused by the steady formation of C-S-H on the pre-formed dense microstructure of UHPC. This is plausible, since the formation was dominated by OPC hydration (rather than pozzolanic reaction) at later ages. which should involve the micro-expansion of existing hydration products to secure space for subsequent hydration;
2. In terms of the degree of hydration of UHPC, the QXRD and TGA showed similar results. At 28 days, both QXRD and NMR indicated that a significant amount of silica fume was left, which would produce a physical filling effect on the UHPC. The NMR also showed the evolution of the nanostructure of C-S-H, as the MCL of C-S-H increased over time. This structural change was not considered in the C-S-H model adopted in the PONKCS method. Therefore, the variation of crystallinity of C-S-H or the structural difference of C-S-H in regular OPC hydration and UHPC can produce an error when quantifying C-S-H using the PONKCS method;
3. This study was able to quantify the two main amorphous phases (i.e., silica fume and C-S-H) in UHPC. During the heat treatment of UHPC, the consumption of silica fume was dominant, as shown by the more active pozzolanic reaction. The evolution of the quantitative content of C-S-H was well correlated with the development of strength. At a very early stage of heat treatment, QXRD underestimated the content of silica fume; the dispersed silica fume with a small contribution of XRD peak intensity was consumed first during the heat treatment.

## Figures and Tables

**Table 1:** Mix proportions of specimens by weight ratio.

Mixture	OPC	Silica Fume	Quartz Powder	Silica Sand	Water	Superplasticizer
UHPC	1000	250	350	1100	225	40

Solid-state superplasticizer was used.

**Table 2:** Deconvolution results for ^29^Si MAS NMR spectra, wt.%.

Specimen	Q^0^ −71 ppm	Q^0^ −74 ppm	Q^1^ −79 ppm	Q^2^(1Al) −81 ppm	Q^2^ −85 ppm	Q^4^ −110 ppm	Al/Si	MCL
**UHPC_1day**	40.00	31.70	0.90	0	0	27.40	0	2.00
**UHPC_2days**	26.93	16.97	5.66	6.18	29.95	14.31	0.074	15.86
**UHPC_4days**	24.38	19.56	5.47	9.04	28.87	12.68	0.10	17.51
**UHPC_28days**	24.19	17.25	3.33	11.90	30.47	12.88	0.13	31.02
